# Evaluation of cardiovascular risk using smartwatch-measured pulse wave velocity in China: a nationwide cross-sectional study

**DOI:** 10.3389/fcvm.2026.1865667

**Published:** 2026-09-28

**Authors:** Luyan Wang, Ningling Sun, Hongyi Wang, Yang Xi, Fan Yang, Yuanyuan Chen, Jing Liu

**Affiliations:** Department of Hypertension, Peking University People’s Hospital, Beijing, China

**Keywords:** arterial stiffness, cardiovascular disease, carotid-femoral pulse wave velocity, hypertension, smartwatch

## Abstract

**Background:**

Cardiovascular and cerebrovascular diseases, driven by arteriosclerosis, are China's leading causes of death. Smartwatch-measured pulse wave velocity (SW-PWV) is an algorithm-derived estimate of carotid-femoral pulse wave velocity (cfPWV) generated using a built-in machine-learning algorithm. A previous validation study showed good agreement between smartwatch-estimated cfPWV and Complior Analyse measurements, with a concordance correlation coefficient of 0.9045 and a mean absolute error of 0.66 m/s.

**Objective:**

This study aimed to describe the distribution of smartwatch-measured pulse wave velocity (SW-PWV) according to age, sex, and body mass index (BMI), and to investigate its cross-sectional associations with cardiovascular risk factors and established cardiovascular disease (CVD).

**Methods:**

This cross-sectional study analyzed 165,549 users who completed at least one valid SW-PWV measurement using compatible Huawei smartwatches between December 2020 and August 2022. Analyses of cardiovascular risk factors and CVD were restricted to 35,402 users who completed the “Vascular Health” app questionnaire, in which physician-diagnosed conditions were self-reported according to predefined criteria. A threshold of 10 m/s, adapted from guideline recommendations for cfPWV, was explored for SW-PWV. Spearman's correlation, Kruskal–Wallis test, multiple regression, and Mann–Whitney *U*-test were used for analysis. Data were reported as median (interquartile range) or mean ± standard deviation.

**Results:**

SW-PWV correlated positively with age, male sex, and BMI. After age- and BMI-stratification, SW-PWV increased progressively across all cohorts. Average SW-PWV was significantly higher in males than in females. SW-PWV was significantly elevated in participants with hypertension, diabetes, dyslipidemia, coronary heart disease, stroke, and carotid plaque, with hypertension and carotid plaque exhibiting the strongest correlations. SW-PWV demonstrated moderate discrimination for prevalent CVD (area under the curve = 0.71). When applying the guideline-recommended cfPWV threshold of 10 m/s to SW-PWV, sensitivity was 13.6% and specificity was 96.7%.

**Conclusion:**

In this cross-sectional study, SW-PWV was associated with age, sex, and BMI, with SW-PWV ≥10 m/s linked to a higher prevalence of cardiovascular risk factors and CVD. These findings suggest that the guideline-derived 10 m/s threshold of cfPWV might serve as a preliminary or exploratory threshold reference for SW-PWV, though direct validation against conventional cfPWV and prospective longitudinal studies are required before clinical implementation.

## 1 Introduction

Cardiovascular and cerebrovascular diseases are the leading causes of death among urban and rural residents in China (1). These diseases share a common pathogenesis known as arteriosclerosis, which is characterized as thickening and hardening of arterial walls (2). Arteriosclerosis is an independent risk factor for many cardiovascular diseases (CVDs), such as hypertension, dyslipidemia, coronary heart disease, stroke, and carotid plaque (3–8). When arterial stiffness increases, the pulse pressure will be elevated, aggravating the left ventricular afterload and leading to various CVDs (9–11). Nevertheless, arteriosclerosis can be asymptomatic over years or decades until signs and symptoms appear, i.e., once the arterial walls (including the aorta's) undergo a remodeling process that entails elastin degradation and collagen deposition (12). The risk factors for arteriosclerosis include age, dyslipidemia, diabetes mellitus, and hypertension (13–15), which are all risk factors for CVDs, but their exact contribution to arterial stiffness remain to be investigated in relation to each other due to the complicated pathogenesis of arteriosclerosis.

Pulse wave velocity (PWV) refers to the conduction velocity of the pressure wave along the wall of the great arteries produced by the ejection of each beat of the heart (16). PWV is closely related to the elasticity of arteries, and the propagation of pulse waves in the arterial system accelerates due to the reduced elasticity of arteries. The geometric characteristics of the blood vessels, blood density, and other factors can reflect the changes in arterial function, and PWV increases with age under normal circumstances (16). In general, the normal value of PWV is <9 m/s in adults <45 years of age and <10 m/s in adults >45 years old. Higher PWV indicates more severe arteriosclerosis, with PWV >13 m/s indicating coronary artery disease (16, 17). Currently, carotid-femoral PWV (cfPWV) is widely accepted and recommended as the gold standard for arterial stiffness evaluation and cardiovascular risk assessment (18–20). cfPWV is described as the pulse wave assessed at carotid and femoral artery sites, and the transmission time and distance between the two sites are used to calculate the cfPWV (21). An increase in cfPWV indicates arterial stiffness aggravation (18). cfPWV is also an index of coronary heart disease (22) and carotid plaque (23). Among the risk factors of CVDs, aging and hypertension are independently associated with cfPWV (24). Many devices have emerged to assess cfPWV, such as SphygmoCor, PulsePen, SphygmoCor Xcel, Complior, Aortic, and Vicorder (21). Nevertheless, due to the need for specialized equipment, availability, training requirements, high cost, and complex operation, the devices used for cfPWV testing are unsuitable for daily measurements by individuals or families, bringing difficulties for the long-term monitoring of chronic diseases and early detection in healthy individuals. Even though other methods are available to assess arterial stiffness, such as aortic PWV, finger-toe PWV, brachial-ankle PWV, and cardiac-ankle PWV, all these methods rely on medical devices. Hence, convenient and dependable methods are needed to evaluate arterial stiffness.

With the rapid development of mobile health (mHealth) technologies, wearable devices have become increasingly important tools for remote cardiovascular monitoring and population health management (25–27). Smartwatches have emerged as widely adopted wearable digital health devices. Smartwatches can now take spot readings of the electrocardiogram (ECG), pulse, and other information and have been increasingly applied to monitor heart rate, detect atrial fibrillation, and assess other cardiovascular health metrics (28–30). Smartwatches use light-emitting diodes (LEDs) and optical sensors to acquire photoplethysmographic (PPG) signals by detecting changes in peripheral blood volume. The ECG and PPG signals acquired by the smartwatch are automatically processed by its built-in algorithm to estimate a carotid–femoral PWV (cfPWV)-like parameter that reflects arterial stiffness (31). In a previous validation study, 347 volunteers aged 18–80 years underwent cfPWV measurements using both a Huawei smartwatch and Complior Analyse, which served as the reference method. Smartwatch-estimated cfPWV and Complior-measured cfPWV were assessed alternately in sitting and supine positions, with nine paired measurements obtained from each participant at 60-second intervals. Agreement and measurement accuracy were evaluated using the concordance correlation coefficient, mean error, mean absolute error, and Bland–Altman analysis. Analyse measurements showed good agreement, with a concordance correlation coefficient of 0.9045 (95% CI, 0.8853–0.9206), a mean error of 0.046 ± 0.92 m/s, 95% limits of agreement ranging from −1.77 to 1.86 m/s, and a mean absolute error of 0.66 m/s (95% CI, 0.59–0.73) (31). This approach may facilitate convenient, repeated, and large-scale assessment of arterial stiffness outside conventional clinical settings. Nevertheless, unlike conventionally measured cfPWV, SW-PWV is based on wrist-derived single-site signals and algorithmic estimation rather than direct carotid–femoral measurements. Moreover, large-scale data on factors associated with SW-PWV remain limited. Therefore, building on the measurement approach validated by Sun et al., this study aimed to describe the distribution of SW-PWV according to age, sex, and BMI, and to assess its cross-sectional associations with cardiovascular risk factors and established CVD.

## 2 Materials and methods

### 2.1 Study population

This cross-sectional study included smartwatch users from 34 provinces and cities in China who performed SW-PWV measurements between December 2020 and August 2022. The compatible smartwatches were commercially available devices purchased and owned by the users; no devices were provided by the investigators, and no active recruitment was conducted. All users who performed SW-PWV measurements during the study period and consented to the research use of their data were considered for inclusion. No *a priori* sample size calculation was performed because all eligible users within the specified study period were included. Before analysis, the collected records underwent data cleaning to exclude users with implausible age or anthropometric information, modified user information, or outlying SW-PWV values. Completion of the health questionnaire through the smartphone-based “Vascular Health” app was voluntary. Users who completed the questionnaire provided additional information on hypertension, diabetes, dyslipidemia, coronary heart disease, stroke, and carotid artery plaque. These questionnaire data were used for analyses of the associations between SW-PWV and cardiovascular risk factors or CVD. Only users with complete questionnaire data were included in analyses involving cardiovascular risk factors and CVD. Users with incomplete questionnaire data were excluded from these disease-related analyses.

### 2.2 Ethical considerations

This study was approved by the ethics committees of Peking University People's Hospital (2020PHB263-01). Each user was required to fill out an electronic informed consent form before taking their first arterial stiffness measurement with the watch and consent to participate in the study and provide data. All data were anonymized once encoded. The individuals received no compensation or incentive.

### 2.3 Data quality control

Data cleaning performed sequentially. First, users with implausible demographic or anthropometric information were excluded, including age <18 (because the body is still under development, affecting PWV analysis) or >90 years (because of the small proportion of the population they represent and because of the numerous comorbidities than can affect the results), height <130 or >210 cm (to exclude outliers and the possibility of data entry error), weight <30 or >200 kg (to exclude outliers and the possibility of data entry error), or BMI <10 or >50 kg/m^2^ (to exclude outliers and the possibility of data entry error). Second, outlier screening of repeated historical PWV measurements was performed at the individual level. All historical PWV measurements were grouped by person ID, and an individual-specific mean and standard deviation (SD) were calculated for each user. A measurement falling outside the individual-specific interval of mean ± 3 SD was considered an outlier. If a user had at least one such outlying measurement, the entire user record was excluded to ensure the strict physiological stability and reliability of the individual's baseline data for this cross-sectional analysis. Thus, the ±3 SD criterion was applied within individuals rather than to the overall SW-PWV distribution, and no single population-level numerical cut-off was used for outlier exclusion. Third, users whose demographic or anthropometric information had been subsequently modified were excluded. The process of data cleaning is shown in Figure 1.

**Figure 1:**
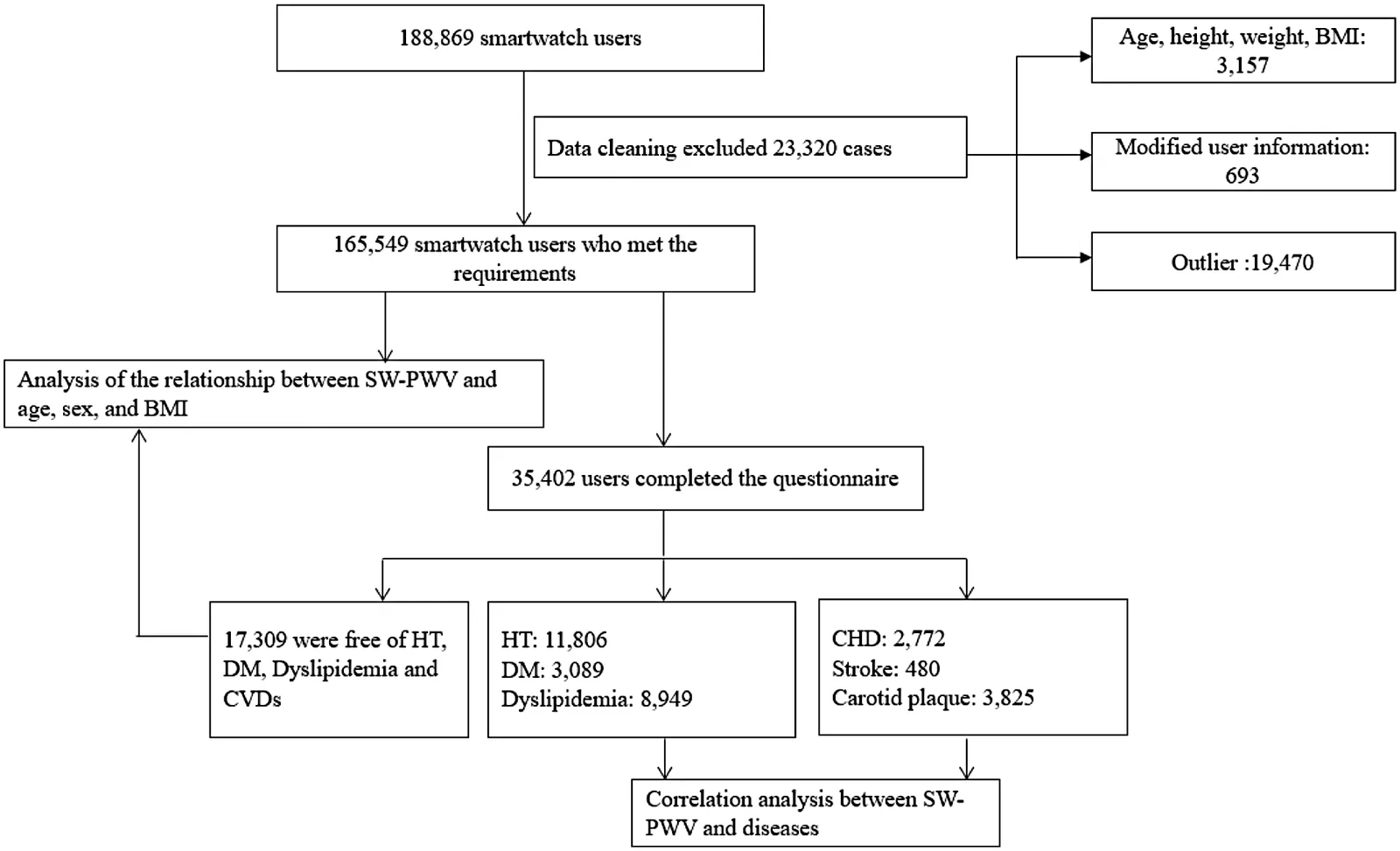
Enrollment process.

### 2.4 Data collection of SW-PWV from smartwatch

In this study, four commercially available smartwatches that support PWV detection were used to access the data, including GT2 Pro ECG, GT3 Pro, WATCH D, and WATCH 3 Pro new (all manufactured by Huawei Device Co., Ltd., Shenzhen, China). The Vascular Health APP is a mobile phone application that is compatible with smartwatches and was developed by Huawei. The “Vascular Health” APP contains detailed operational instructions and a description of the detection method. The APP also informed users that the vascular health assessment was not intended for individuals younger than 18 years, pregnant women, individuals with implanted cardiac pacemakers, or those with diagnosed severe arterial stenosis or cardiac arrhythmias.

The research team collaborated with Huawei in the previous validation of the smartwatch-based PWV method and in the scientific evaluation of the Vascular Health APP. Huawei provided the investigators with technical explanations regarding the general principles and input variables used in the SW-PWV estimation algorithm. The investigators did not have access to the proprietary source code or the full internal implementation of the algorithm. Participants were required to read these instructions carefully within the APP before performing the measurement to ensure data accuracy. They were instructed to perform the measurement while seated and quiet in a temperature-controlled indoor environment and to wear the smartwatch snugly, with the strap adjusted to allow the little finger to be inserted beneath it without excessive tightness. First, the individuals remained seated for 10 min before testing. Afterward, the smartwatch wearers entered the “Vascular Health” app on their compatible mobile phones and activated the corresponding “Vascular Health” on their smartwatches to start the measurement. During measurement, the individuals wore the smartwatch on their left hand and touched the collector with the right index finger for 30 s. The SW-PWV measurements were then recorded on the “Vascular Health” APP on the mobile phone and shared with the investigators.

The smartwatch simultaneously acquires electrocardiographic (ECG) and photoplethysmographic (PPG) signals during the measurement. The ECG R wave provides the timing of ventricular depolarization, whereas the PPG signal detects the arrival of the peripheral pulse wave at the wrist. The time interval between the ECG R wave and the foot of the PPG waveform is used to estimate pulse transit time (PTT), which reflects the propagation time of the arterial pulse wave. PTT was calculated as follows: PTT = T_PPG_foot_−T_ECG_R_, where T_PPG_foot_ represents the time point at which the foot of the PPG waveform is detected and T_ECG_R_ represents the time point of the ECG R-wave peak. PWV was subsequently estimated as follows: PWV = L/PTT, where L represents the estimated effective arterial path length. Because the smartwatch cannot directly measure the arterial path length, L was estimated using anthropometric parameters incorporated into the built-in algorithm. To improve estimation accuracy, the algorithm further integrates demographic characteristics, including age, sex, and BMI, together with calibration based on previous validation studies. Measurements that do not satisfy the built-in signal quality criteria are automatically rejected by the smartwatch algorithm.

Only the first valid measurement was used in the present study to avoid selectively choosing the most favorable value from multiple measurements. Measurements that did not meet the quality requirements of the app's automated signal-quality assessment were considered invalid and were not included in the analysis.

### 2.5 Smartwatch user general information collection

Sex, age, height, and weight information were self-reported by users through the “Vascular Health” APP supported by the mobile phone. Body mass index (BMI) was calculated as weight in kilograms divided by the square of height in meters (kg/m²).

### 2.6 Collection of disease information

The questionnaires were administered through the “Vascular Health” mobile APP to investigate the diagnosis of CVDs among smartwatch users, including hypertension, diabetes, dyslipidemia, coronary heart disease, stroke, and carotid artery plaque. Information on these conditions was self-reported through the questionnaire based on users’ recollection of previous physician diagnoses and clinical examinations. Specific diagnostic criteria were defined for each disease category as follows. Hypertension: defined as a physician's diagnosis of systolic blood pressure ≥140 mmHg and/or diastolic blood pressure ≥90 mmHg on three or more occasions on different days. Diabetes mellitus: diagnosed by a clinician when blood glucose level is higher than the diagnostic criteria. Dyslipidemia: confirmed by a clinician after a blood lipid test. Coronary heart disease: patients diagnosed with coronary heart disease by exercise test, coronary CT, coronary angiography, previous myocardial infarction, coronary artery intervention, or coronary artery bypass grafting. Stroke: patients diagnosed with stroke based on clinical findings or head CT or MRI. Carotid artery plaque: patients with carotid artery plaque detected by carotid artery ultrasound examination.

### 2.7 Age, BMI, and comorbidity grouping

The individuals were divided into six groups based on age (years): <30, 30–39, 40–49, 50–59, 60–69, and ≥70 years. The BMI groups were categorized as <18.5 (low), 18.5–24 (normal), 24–28 (overweight), and ≥28 (obesity) kg/m^2^. The individuals were grouped according to the number of comorbidities as 0–1 vs. ≥2 comorbidities.

### 2.8 Statistical analysis

The Python programming language (3.8.10) was used to conduct statistical analysis. The normality of the data was first analyzed using the Kolmogorov–Smirnov and Jarque–Bera tests, and the data were described as the median (interquartile range). Categorical data are shown as *n* (%) and analyzed using the chi-squared test. Spearman's correlation coefficient was used to evaluate the correlations between SW-PWV and continuous variables (including age and BMI). The point-biserial correlation and Mann–Whitney *U*-test were utilized to evaluate the association and differences between SW-PWV and sex. Multivariable correlation analysis was performed to evaluate the associations between SW-PWV measurements and potentially associated factors (including sex, age, and BMI) across distinct age groups. The correlation coefficients were classified as.00 to.19 for very weak or negligible correlation,.20 to.39 for weak correlation,.40 to.59 for moderate correlation,.60 to.79 for strong correlation, and.80 to 1.00 for very strong correlation. The Mann–Whitney *U*-test was used to compare SW-PWV between two groups, including sex and comorbidity groups, whereas the Kruskal–Wallis test was used to compare SW-PWV across the six age groups and four BMI groups. To visualize trends in comorbidity and CVD prevalence, SW-PWV measurements were categorized into 0.5 m/s intervals ranging from 6.0 to 12.5 m/s. Relative risks (RRs) and corresponding 95% confidence intervals were calculated using 2 × 2 contingency tables to compare the prevalence of SW-PWV ≥10 m/s between participants with each cardiovascular risk factor or CVD and those without the corresponding condition as the reference group. These RR estimates were unadjusted. Multiple ordinary least-squares (OLS) linear regression analysis was used to analyze the multivariable associations of SW-PWV with age, sex, and BMI. In the regression model, SW-PWV was the dependent variable, and the independent variables included age, sex, and BMI. The selection of these variables was based on their known association with arteriosclerosis (theoretical basis) and their significance in the univariable analyses (statistical basis). All independent variables were included in the model at the same time, and no stepwise regression method was used. Receiver operating characteristic (ROC) curve analysis was performed to evaluate the ability of SW-PWV to discriminate between users with and without prevalent CVD. The area under the ROC curve (AUC) was calculated. Sensitivity and specificity were evaluated at the guideline-derived threshold of 10 m/s, and the exploratory optimal threshold was determined by maximizing the Youden index.

In addition, age-stratified ROC analyses were performed to evaluate the ability of SW-PWV to discriminate prevalent hypertension within each prespecified age group. For each age group, the AUC and its 95% confidence interval were calculated, and the optimal probability cutoff was determined by maximizing the Youden index, with the corresponding sensitivity and specificity reported. The statistical significance of each AUC was also assessed. The threshold value of statistical significance was set at *P* < .05.

## 3 Results

### 3.1 General information of the individuals

This study collected data from 188,869 individuals who completed the SW-PWV detection using smartwatches across 34 provinces and cities in China from December 2020 to August 2022. Of these, 3,157 records were excluded because of implausible age or anthropometric data, defined as age <18 or >90 years, height <130 or >210 cm, weight <30 or >200 kg, or body mass index (BMI) < 10 or >50 kg/m^2^. A further 19,470 users were excluded because at least one of their repeated historical PWV measurements fell outside the individual-specific mean ± 3 SD interval, and 693 records were excluded because the users' profile information had been modified (Figure 1). Before individual-level outlier screening (*n* = 185,712), SW-PWV had a mean of 8.03 ± 0.94 m/s, a median of 7.76 m/s (IQR, 7.45–8.38), and a range of 5.48–15.35 m/s; 8,145 users (4.39%) had SW-PWV ≥10 m/s (Supplementary Table S1 and Supplementary Figure S1A). After outlier screening (*n* = 166,242), the corresponding values were 8.04 ± 0.95 m/s, 7.79 m/s (IQR, 7.46–8.40), and 5.49–15.00 m/s, respectively, with 7,524 users (4.53%) having SW-PWV ≥10 m/s (Supplementary Table S1 and Supplementary Figure S1B). Among the 19,470 users excluded by the outlier-screening criterion, the mean SW-PWV was 7.93 ± 0.85 m/s, the median was 7.66 m/s (IQR, 7.44–8.20), and the range was 5.52–14.52 m/s; 621 users (3.19%) had SW-PWV ≥10 m/s (Supplementary Table S1 and Supplementary Figure S1C). Overall, the SW-PWV distribution changed only minimally after outlier screening.

After data cleaning, 165,549 users remained in the study. Among them, 35,402 (21.4%) completed the health questionnaire, while 130,147 (78.6%) did not provide complete questionnaire data. The average age of the individuals was 41.64 ± 11.81 years old. Among them, 88.03% were males (145,740 individuals), while females accounted for 11.97% (19,809 individuals). The average height, weight, BMI, and SW-PWV were 172.04 ± 6.85 cm, 75.29 ± 13.64 kg, 25.34 ± 3.75 kg/m^2^, and 7.79 (7.46–8.40) m/s respectively (Table 1). A threshold of 10 m/s, derived from the 2018 ESC/ESH guideline recommendation for cfPWV (19), was explored as a reference value for SW-PWV in the present study.

**Table 1:** General information of study individuals (*n* = 165,549).

Index	Value
Overall study population (*n* = 165,549)	
Sex, male/female, *n* (%)	145,740 (88.03%)/19,809 (11.97%)
Age, years	41.64 ± 11.81
Height, cm	172.04 ± 6.85
Weight, kg	75.29 ± 13.64
BMI, kg/m^2^	25.34 ± 3.75
SW-PWV, m/s, median (P25-P75)	7.79 (7.46-8.40)
Questionnaire subgroup (*n* = 35,402)	
Hypertension, *n* (%)	11,806 (33.35)
Diabetes, *n* (%)	3,089 (8.73)
Dyslipidemia, *n* (%)	8,949 (25.28)
CHD, *n* (%)	2,772 (7.83)
Stroke, *n* (%)	480 (1.36)
Carotid plaque, *n* (%)	3,825 (10.80)

BMI, body mass index; SW-PWV, smartwatch pulse wave velocity; CHD, coronary heart disease.

### 3.2 Correlation analysis of SW-PWV with age and sex

Aging and sex were reported to be closely related to arterial stiffness development (32, 33). There was a moderate-to-strong positive correlation between SW-PWV and age (*r* = .6323, *P* < .0001) (Supplementary Table S2). Taking age groups spanning 10 years, the individuals were divided into six age groups. As shown in Table 2 and Figure 2A, SW-PWV increased with age. There were significant differences between different age groups (Kruskal–Wallis test statistic = 80,553.3, *P* *<* .0001). Furthermore, the correlation analysis of SW-PWV with sex revealed a close correlation, but Spearman's correlation coefficient (*r* = .1856, *P* *<* .0001) was weaker than for age. Interestingly, the results showed that the average SW-PWV of males (median SW-PWV (P25-P75) = 7.83 (7.50–8.39) m/s) was higher than that of females (median SW-PWV (P25-P75) = 7.27 (6.84–8.64) m/s). These results indicated that SW-PWV was closely correlated with age and sex, with the average SW-PWV of males being higher than in females.

**Table 2:** The difference of SW-PWV in different age groups after sex stratification (median, P25-P75).

Age category (years)	*n*	SW-PWV	Sex	*n*	SW-PWV	*P*
<30	24,879	7.33 (7.17–7.64)	Male	22,094	7.36 (7.23–7.70)	<.001
			Female	2,785	6.58 (6.34–6.82)	
30–39	52,905	7.60 (7.43–7.96)	Male	47,809	7.66 (7.49–7.99)	<.001
			Female	5,096	6.88 (6.73–7.13)	
40–49	48,015	7.66 (7.49–8.06)	Male	43,400	7.74 (7.53–8.11)	<.001
			Female	4,615	7.19 (6.97–7.36)	
50–59	27,697	8.96 (8.33–9.32)	Male	23,786	9.04 (8.41–9.35)	<.001
			Female	3,911	8.52 (7.96–8.98)	
60–69	8,626	9.77 (9.22–10.29)	Male	6,467	9.76 (9.25–10.29)	<.001
			Female	2,159	9.85 (9.04–10.28)	
≥70	3,427	11.28 (10.70–11.98)	Male	2,184	11.30 (10.74–11.99)	<.001
			Female	1,243	11.22 (10.61–11.95)	

SW-PWV, smartwatch pulse wave velocity.

**Figure 2:**
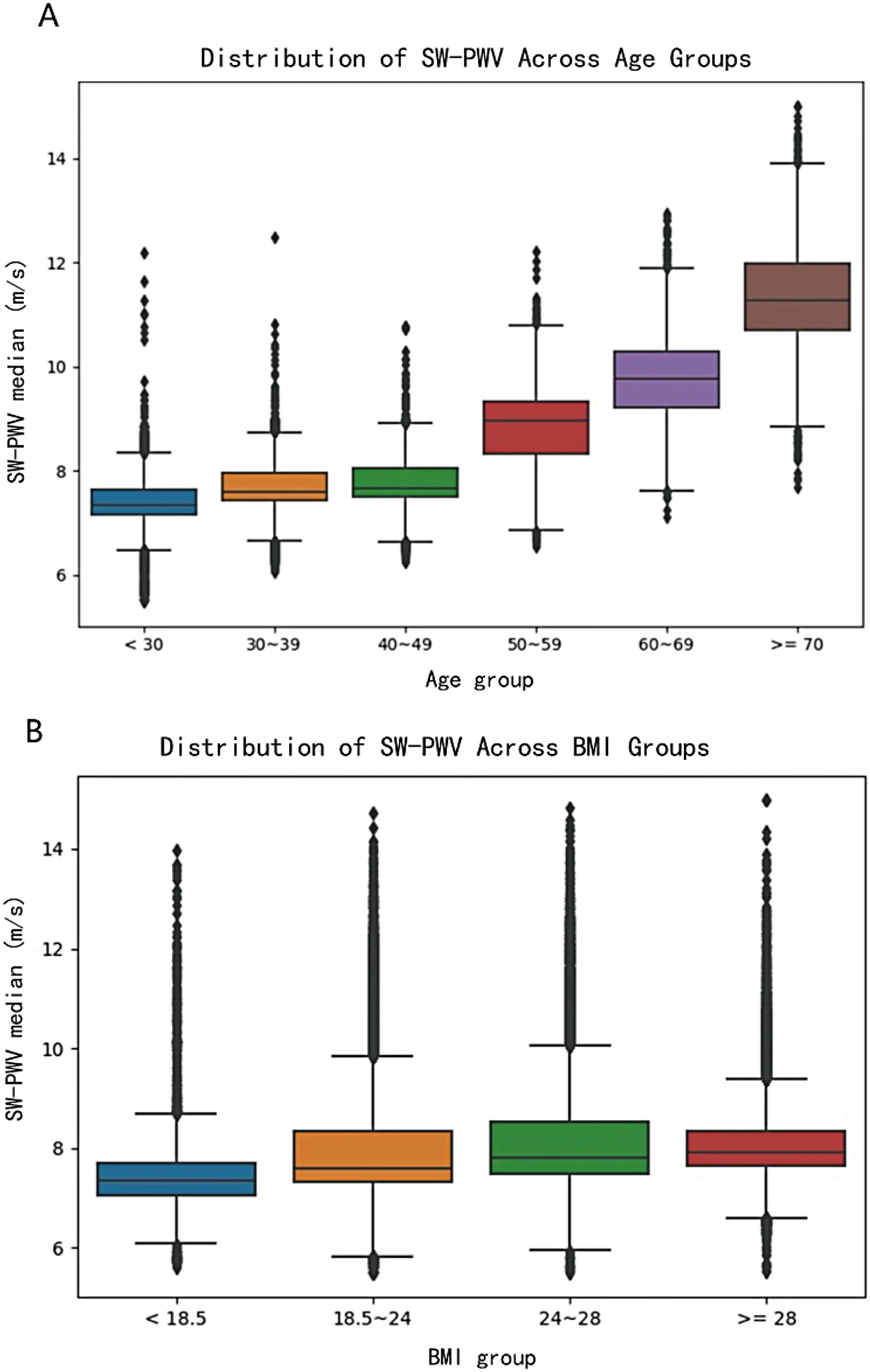
Associations of SW-PWV with age and BMI. **(A)** Distribution of SW-PWV across six age groups. **(B)** Distribution of SW-PWV across four BMI groups. Differences among groups were assessed using the Kruskal–Wallis test. Box plots show the median (center line), 25th percentile (lower boundary), and 75th percentile (upper boundary). BMI, body mass index; SW-PWV, smartwatch-measured pulse wave velocity.

### 3.3 Correlation analysis between SW-PWV and BMI

Previous studies have shown that elevated BMI is associated with increased arterial stiffness (34). There was a significantly positive correlation between SW-PWV and BMI (*r* = .1981, *P* *<* .0001) (Supplementary Table S2). The BMI of all individuals was separated into four groups, and the Kruskal–Wallis test analysis was performed. There were significant differences in SW-PWV among the four groups of BMI, and SW-PWV was increased along with BMI increasing (Figure 2B).

### 3.4 Variation in SW-PWV according to age, sex, and BMI

We examined the associations of SW-PWV with age, sex, and BMI across different age groups. SW-PWV was correlated with age, sex, and BMI in different age groups (Table 3). The association between sex and SW-PWV was relatively stronger in younger participants and became weaker in older age groups. The association between age and SW-PWV was weaker in the middle age strata but became more pronounced in participants aged ≥50 years, particularly in those aged 60–69 years. BMI was positively associated with SW-PWV across all age groups, although the strength of the association varied by age.

**Table 3:** Relationship between SW-PWV and age, sex, and BMI in various age groups.

Age category (years)	*n*	Sex		Age		BMI	
		*r*	*P*	*r*	*P*	*r*	*P*
<30	24,879	.5033	<.001	.3085	<.001	.2858	<.001
30–39	52,905	.4708	<.001	.0985	<.001	.3657	<.001
40–49	48,015	.4267	<.001	.0198	<.001	.2676	<.001
50–59	27,697	.2021	<.001	.4254	<.001	.2907	<.001
60–69	8,626	.0347	.001	.7212	<.001	.1458	<.001
≥70	3,427	.0438	.010	.5974	<.001	.0867	<.001

BMI, body mass index; SW-PWV, smartwatch-measured pulse wave velocity.

We also conducted multiple linear regression analyses of SW-PWV with age, sex, and BMI as variables and established a regression model. The model explained 60.9% of the variance in SW-PWV (R^2^ = 0.609, *P* < 0.01). Age, sex, and BMI were all statistically associated with SW-PWV in the model (all *P* < 0.001; Table 4).

**Table 4:** OLS regression results.

Index	Coef	Std err	*t*	*P*|t|	CI [0.025–0.975]
Intercept	4.6175	0.011	401.944	0.000	4.595–4.640
Age	0.0623	0.000	502.362	0.000	0.062–0.063
Sex	0.4584	0.005	98.942	0.000	0.449–0.467
BMI	0.0168	0.000	42.141	0.000	0.016–0.018

BMI: body mass index.

### 3.5 Associations of SW-PWV with comorbidities and CVD

We next investigated the associations between SW-PWV and cardiovascular risk factors, including hypertension, diabetes, and dyslipidemia, which are three widely accepted risks for CVDs that might progress to severe cardiovascular dysfunction diseases (35, 36). In this study, 35,402 smartwatch wearers completed the questionnaire and were enrolled, with an average age of 44.18 ± 11.84. Males comprised 93.72% of the individuals, and the mean BMI was 25.50 ± 3.59. Figure 3A shows that with the rise of SW-PWV measurements, the proportion of hypertension, diabetes, and dyslipidemia progressively increased. SW-PWV showed significant correlations with hypertension, diabetes, and dyslipidemia, with *r* = .28, *r* = .19, and *r* = .17, respectively. Moreover, *r* = .32 was observed for two or more diseases (*P* *<* .0001). By conducting the same correlation analysis in various age cohorts, the results showed that hypertension, dyslipidemia, and two or more diseases were even more significantly correlated with SW-PWV in all age groups under 60 years old. In addition, diabetes was significantly correlated with SW-PWV in age groups between 30 and 70 years old, as shown in Table 5. Notably, the overall correlation between SW-PWV and hypertension was markedly attenuated after age stratification, with age-specific correlation coefficients ranging from 0.0336 to 0.1656. Age-stratified ROC analyses further showed limited discrimination of prevalent hypertension, with AUCs ranging from 0.508 to 0.589 across age groups (Supplementary Table S3 and Supplementary Figure S2). The AUCs were not significantly different from 0.5 in participants aged 60–69 years and ≥70 years, indicating that SW-PWV did not show statistically significant discrimination of prevalent hypertension in these two age groups.

**Figure 3:**
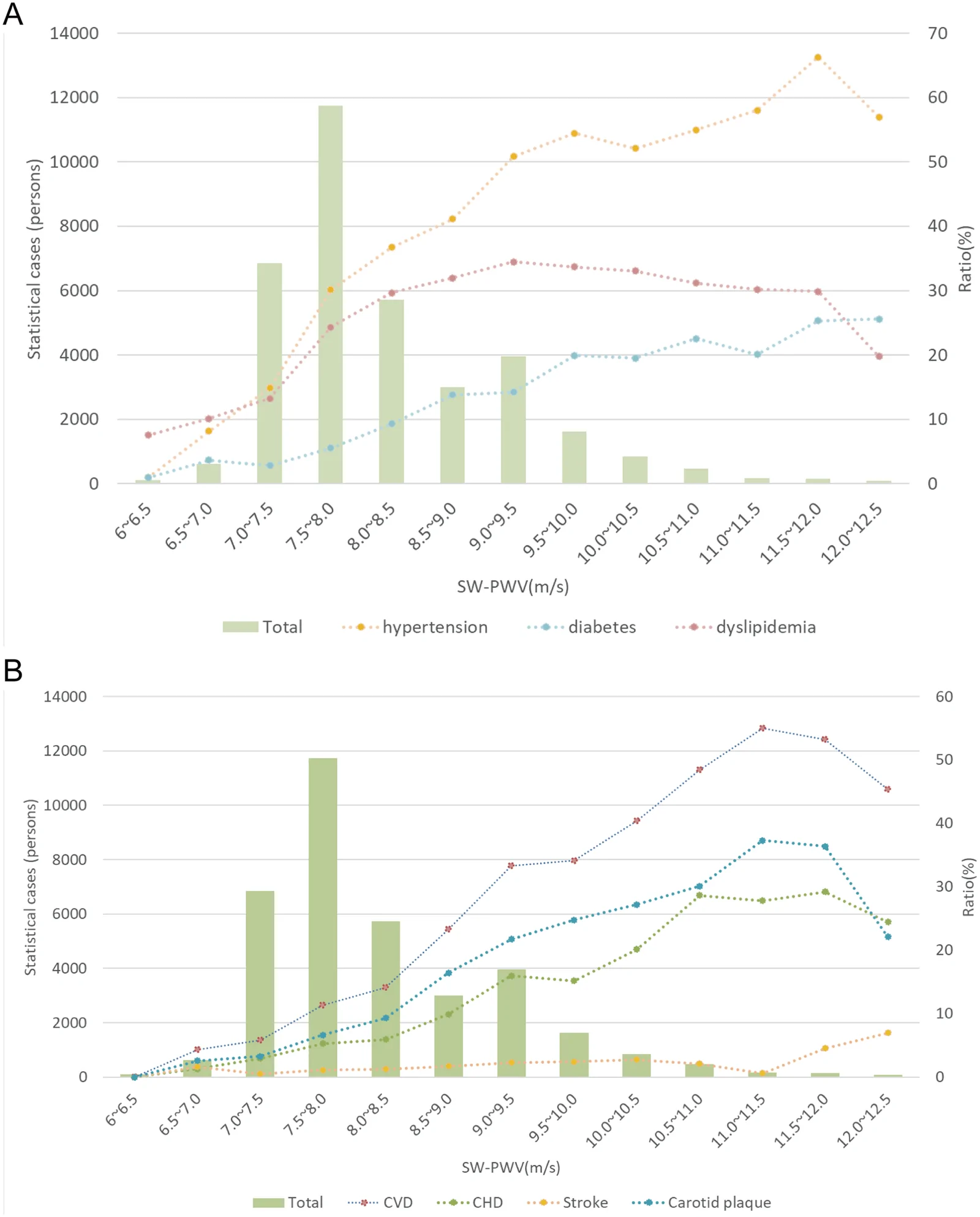
Associations of SW-PWV with cardiovascular risk factors and CVD. **(A)** Relationship between SW-PWV values and hypertension, diabetes, and dyslipidemia. The bar charts showed the proportion of individuals in different ranges of SW-PWV values. The broken lines showed the proportion of patients with hypertension, diabetes, and dyslipidemia at different ranges of SW-PWV. Yellow: hypertension; Blue: diabetes; Red: dyslipidemia. **(B)** Relationship between SW-PWV values and coronary heart disease, stroke, carotid plaque, and total CVD. The bar charts showed the proportion of individuals in different ranges of SW-PWV values. The broken lines showed the proportion of patients with coronary heart disease, stroke, carotid plaque, and total CVD at different ranges of SW-PWV levels. Red: CVD; Green: CHD; Yellow: stroke; Blue: carotid plaque. CHD, coronary heart disease; CVD, cardiovascular disease; SW-PWV, smartwatch-measured pulse wave velocity.

**Table 5:** Correlation analysis between comorbidities and SW-PWV after age stratification.

Age category (years)	Hypertension		Dyslipidemia		Diabetes		Two diseases or more	
	*r*	*P*	*r*	*P*	*r*	*P*	*r*	*P*
<30	.0985	<.001	.0658	<.001	.0137	.37	.1157	<.001
30–39	.1656	<.001	.1076	<.001	.0972	<.001	.1807	<.001
40–49	.1426	<.001	.0885	<.001	.0898	<.001	.1522	<.001
50–59	.1147	<.001	.024	.025	.0986	<.001	.0975	<.001
60–69	.0411	.041	−.0083	.683	.0634	.002	.0589	.004
≥70	.0336	.38	−.0493	.20	.08	.035	−.008	.834

SW-PWV, smartwatch-measured pulse wave velocity.

Next, a correlation analysis was conducted between SW-PWV and CVD, including coronary heart disease, stroke, and carotid plaque, to study the associations between CVDs and SW-PWV levels. Figure 3B demonstrated that with the increase of SW-PWV values, there were significant increases in the prevalence of coronary heart disease, stroke, and carotid plaque. Specifically, a higher prevalence of CVD was observed among individuals with higher SW-PWV levels. Furthermore, the mean SW-PWV values were separately recorded for different populations suffering or not from various CVDs. Compared with those without CVD, SW-PWV levels in individuals with CVD were significantly higher (*P* *<* .0001) (Supplementary Table S4). Among the three CVDs, the occurrence of carotid plaque showed the strongest correlation with the SW-PWV measurements, followed by coronary heart disease. Despite stroke demonstrating the weakest SW-PWV correlation, its occurrence still significantly correlated with the SW-PWV values (*P* *<* .0001). In summary, the prevalence of these three CVDs was positively associated with elevated SW-PWV levels.

Finally, we attempted to stratify the SW-PWV data of the participants according to the cfPWV threshold of 10 m/s recommended in the guideline, to further evaluate its association with cardiovascular risk factors and CVD. Individuals were divided into two groups based on their SW-PWV measurements: the high SW-PWV level group with no less than 10 m/s, and the low SW-PWV level group with less than 10 m/s. The proportion of individuals suffering from the total number of CVDs was analyzed between the two groups. Supplementary Table S5 shows a significant difference in the ratio of individuals with 0, 1, or 2 or more CVDs among different SW-PWV level groups. In the low SW-PWV level group, 84.76% of the individuals were free from CVDs, 12.69% had one type of CVDs, and only 2.56% suffered from two or more types of CVDs. In contrast, in the high SW-PWV level group, only 54.43% were free from CVDs. In comparison, a higher proportion of 34.77% were afflicted by one type of CVDs, and 10.80% exhibited two or more types of CVDs, indicating a comparatively higher prevalence of CVDs within this group. The chi-squared test results suggested a statistically significant difference between the two SW-PWV level groups (*χ*^2^ = 1,184.7599, *P* *<* .0001). ROC analysis yielded an AUC of 0.71 for discriminating between users with and without prevalent CVD. At the threshold of 10 m/s, sensitivity was 13.6% and specificity was 96.7%. The maximum Youden index identified an exploratory optimal threshold of 8.53 m/s, with a sensitivity of 56.5% and a specificity of 76.9% (Supplementary Figure S3). Furthermore, the prevalence of cardiovascular diseases was compared in populations with different SW-PWV levels. As shown in Figure 4A, compared with individuals without hypertension, diabetes, or dyslipidemia, the prevalence of single or multiple comorbidities was significantly higher among individuals with high SW-PWV levels (≥10 m/s). In addition, compared with individuals without CVDs, users with SW-PWV ≥10 m/s had a significantly higher prevalence of single or multiple CVDs (coronary heart disease, stroke, and carotid plaque co-existed), as shown in Figure 4B. Elevated SW-PWV (≥10 m/s) was associated with a higher prevalence of cardiovascular risk factors and CVDs in this cross-sectional analysis.

**Figure 4:**
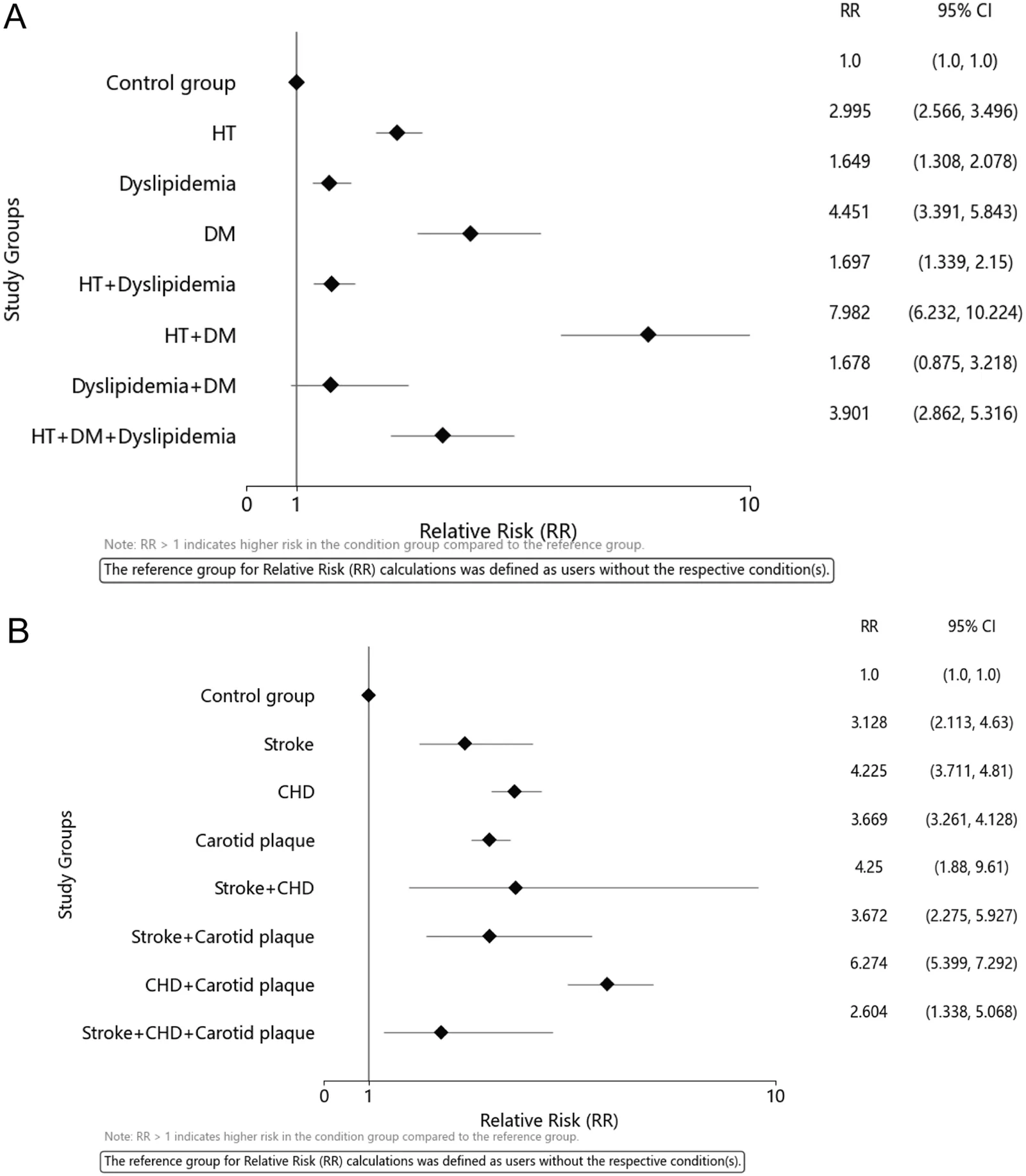
Cross-sectional associations between SW-PWV ≥10 m/s and cardiovascular risk factors or CVD. **(A)** Relative risks (RRs) for SW-PWV ≥10 m/s among individuals with hypertension, diabetes, dyslipidemia, or combinations of these conditions, compared with individuals without hypertension, diabetes, or dyslipidemia. **(B)** Relative risks (RRs) for SW-PWV ≥10 m/s among individuals with coronary heart disease, stroke, carotid plaque, or combinations of these conditions, compared with individuals without coronary heart disease, stroke, or carotid plaque. The reference group for Relative Risk (RR) calculations was defined as users without the respective condition(s). CHD, coronary heart disease; CI, confidence interval; CVD, cardiovascular disease; RR, relative risk; SW-PWV, smartwatch-measured pulse wave velocity.

## 4 Discussion

### 4.1 Principal findings

This study aimed to investigate the association of SW-PWV with demographic characteristics, cardiovascular risk factors, and CVD in a large real-world population. SW-PWV varied according to age, sex, and BMI. Associations between age and SW-PWV were observed not only in older participants but also in younger and middle-aged groups, and the average SW-PWV was higher in males than in females. However, because age, sex, and BMI are incorporated into the SW-PWV estimation algorithm, the observed patterns for these variables should be interpreted as characteristics of the algorithm-derived output rather than as independent biological associations with arterial stiffness. SW-PWV was significantly higher in patients with hypertension, diabetes, dyslipidemia, coronary heart disease, stroke, and carotid artery plaque. Among them, hypertension and carotid artery plaque exhibited the strongest correlation with SW-PWV. As there are currently no studies on the SW-PWV threshold, in this cross-sectional analysis, we attempted to use the cfPWV threshold 10 m/s recommended by the guidelines, to evaluate SW-PWV association with cardiovascular risk factors and CVD. The result showed that SW-PWV ≥10 m/s was associated with a higher prevalence of cardiovascular risk factors and prevalent CVD; however, this threshold should be considered exploratory.

### 4.2 Comparison to prior work

Analyzing the SW-PWV data derived from 165,549 smartwatch users revealed that SW-PWV varied across age groups, with generally higher values in older participants. Similar age-related patterns have been well documented in studies using conventionally measured PWV (37, 38). Age-related variation in SW-PWV was also observed among younger participants. Although this finding cannot independently establish early vascular aging because of the algorithmic dependence on age, previous studies using conventional vascular assessments have suggested that vascular aging may begin in early adulthood (39). Furthermore, SW-PWV was associated with hypertension, diabetes, dyslipidemia, coronary heart disease, stroke, and carotid plaque. SW-PWV ≥10 m/s was associated with a higher prevalence of CVD. The study suggests that SW-PWV may serve as a convenient and promising surrogate marker of arterial stiffness, supported by preliminary reliability evidence from a previous validation study (31). However, the present study did not directly compare SW-PWV with conventionally measured cfPWV or other established arterial stiffness assessments. Therefore, further validation in diverse populations and clinical settings is required before SW-PWV can be considered an established clinical assessment tool.

An important finding of this study was that SW-PWV differed by sex across age groups. The study involved 145,740 male and 19,809 female individuals. Although the number of female smartwatch users was lower than males, it exceeded the minimum requirement of social population statistics by far (23). The study observed an association between SW-PWV and sex of different age groups. Among individuals <60 years old, the SW-PWV of females exhibited significantly lower than in males. SW-PWV was extremely increased in women over 60 years old, reaching levels comparable to or even higher than those in men. Previous studies have reported sex-related differences in arterial stiffness across the life course and have suggested potential contributions from sex hormones, vascular reactivity, and extracellular matrix composition (40–42). However, given that sex is incorporated into the SW-PWV estimation algorithm, the sex-related patterns observed in the present study cannot be attributed independently to underlying vascular biology or hormonal status. Further studies using independently measured cfPWV are needed to clarify the biological basis of these sex-related differences.

Another finding was SW-PWV also varied according to BMI, with generally higher values observed in participants with higher BMI. Previous studies using conventionally measured PWV have reported associations between overweight, obesity, abdominal adiposity, visceral fat, and greater arterial stiffness (43–47). However, the relationship between adiposity and arterial stiffness is complex, and previous studies have reported inconsistent findings that may vary according to age, sex, body composition, and metabolic status (48, 49). Potential mechanisms proposed in previous studies include obesity-related changes in vascular inflammation and adipose tissue signaling, although these mechanisms were not assessed in the present study (50–52).

The correlation analysis between SW-PWV levels and hypertension, diabetes, and dyslipidemia demonstrated a significant increase in SW-PWV accompanied by the occurrence of these three comorbidities, with the most pronounced increase observed in individuals with concurrent hypertension and diabetes. Specifically, SW-PWV levels showed significant associations with individual comorbidities or their combination among individuals <70 years but not in those >70. Previous studies confirmed the close relationship between cfPWV and hypertension, but the conclusions are inconsistent in the studies of diabetes and dyslipidemia. A systematic review of 12 cfPWV-related studies, with data from 26,970 individuals, found that 90% of the studies showed an association between cfPWV and blood pressure, and 52% found an association between cfPWV and diabetes, only 1%–11% of studies have found that cfPWV was associated with dyslipidemia (24). The present study suggested that the associations of SW-PWV with hypertension, diabetes, and dyslipidemia varied across age groups. Age-stratified analyses showed that hypertension was significantly associated with SW-PWV in participants younger than 70 years, whereas the association was no longer statistically significant in those aged ≥70 years. Dyslipidemia was significantly associated with SW-PWV mainly in participants younger than 60 years, while diabetes showed significant associations in most age groups between 30 and ≥70 years. Overall, these findings suggest that the associations between SW-PWV and cardiometabolic comorbidities vary according to age. Age-stratified ROC analyses showed limited discrimination of prevalent hypertension, with AUCs ranging from 0.508 to 0.589 across age groups, indicating that the discriminatory performance of SW-PWV for prevalent hypertension varied across age groups. These age-related differences should also be interpreted in the context of age being incorporated into the SW-PWV estimation algorithm.

The correlation analysis of SW-PWV level and CVD, including coronary heart disease, stroke, and carotid artery plaque, exhibited that the SW-PWV level of individuals with CVD was significantly higher than in individuals without CVD, and the increase in SW-PWV was obvious in individuals with more than two kinds of CVDs. Among all SW-PWV-related CVDs, the elevation in SW-PWV was most obvious in individuals with carotid artery plaque and coronary heart disease. Previous studies consistently confirmed the association of cfPWV with carotid and coronary atherosclerotic diseases, along with related adverse events (53–56). Cohort studies have shown that each 1 m/s increase in cfPWV is associated with an 11% increased risk of carotid plaque, even after adjusting for confounding factors. In addition, cfPWV exhibited a stronger association with carotid plaque than baPWV (23). Patients with confirmed coronary artery disease (CAG) showed significantly faster cfPWV than those with normal coronary angiography, and cfPWV was positively correlated with coronary artery calcification score. The assessment of coronary artery stenosis using cfPWV showed a sensitivity and specificity of 77% and 63%, respectively (5). Consequently, cfPWV is a potential noninvasive screening method for latent coronary artery disease (CAD). Patients with increased cfPWV who underwent coronary angiography had a 34% increased risk of coronary heart disease, and cfPWV was significantly associated with the number and severity of vessels involved in coronary disease and decreased left ventricular diastolic function (57). As for studies related to stroke, the conclusions have not been consistent. Some studies found that cfPWV was significantly increased in patients with intracranial artery stenosis (58), while follow-up studies of patients with acute stroke found that cfPWV was an independent risk factor for all-cause death and cardiovascular events. Nevertheless, cfPWV acceleration cannot independently predict the effects of stroke recurrence and stroke on body function (59). In follow-up studies of older adults over 66 years of age, no association was observed between increased cfPWV and stroke (60). Here, due to the limited number of stroke patients using smartwatches (only 480), the results showed a correlation between stroke and SW-PWV, but it was weaker than the associations observed for coronary heart disease and carotid atherosclerosis.

The European Society of Cardiology recommends a threshold of 10 m/s for assessing arterial stiffness changes in hypertension (19). An increase in cfPWV can serve as an indicator of the risk of future CVDs and is an early assessment parameter for arteriosclerosis in hypertensive patients. In the present data analysis from 35,402 individuals in the questionnaire survey, individuals with cardiovascular risk factors or CVD had a higher prevalence of SW-PWV ≥10 m/s. In the present study, 10 m/s was explored as an exploratory reference threshold for SW-PWV rather than established as a new device-specific cut-off. SW-PWV ≥10 m/s was associated with a higher prevalence of cardiovascular risk factors and prevalent CVD. ROC analysis yielded an AUC of 0.71 for discriminating between users with and without prevalent CVD. At the 10 m/s threshold, sensitivity was 13.6% and specificity was 96.7%, indicating high specificity but limited sensitivity. The maximum Youden index identified an exploratory data-derived threshold of 8.53 m/s, with a sensitivity of 56.5% and a specificity of 76.9%. These findings support further evaluation of SW-PWV thresholds; however, direct validation against cfPWV and prospective longitudinal studies are required before clinical application.

### 4.3 Strengths and limitations

The main strength of the present study is the use of big data from tens of thousands of individuals. In addition, the individuals were from all over China.

There are several limitations of this study. Firstly, the proportion of women among smartwatch users in this real-world dataset was substantially lower, resulting in fewer female than male participants. This imbalance may reflect sex-related differences in smartwatch purchasing, ownership, and usage patterns, including greater adoption of electronic wearable devices among men. However, these factors were not directly assessed in the present study. Consequently, the study population may not be representative of the general population, and selection bias cannot be excluded. Although more than 19,000 women were included, the marked sex imbalance may still limit the generalizability of the findings to women. Therefore, the observed sex differences in SW-PWV should be interpreted cautiously and require confirmation in more sex-balanced populations. Secondly, participation was limited to individuals who owned and actively used compatible smartwatches. This user population may differ from the general population in age distribution, socioeconomic status, health awareness, and digital literacy. However, these differences were not directly assessed in the present study. Because socioeconomic information was not collected, we were unable to evaluate or adjust for this potential source of selection bias. Therefore, the study population may not be fully representative of the general Chinese adult population, particularly individuals with lower socioeconomic status or limited access to wearable technology. Thirdly, although users received standardized instructions regarding the measurement environment, posture, and smartwatch fit, all measurements were self-performed under real-world conditions. Therefore, adherence to these instructions, the exact degree of strap tightness, and variations in ambient and skin temperature could not be independently verified or fully standardized, which may have affected the SW-PWV measurements. In addition, only the first valid SW-PWV measurement from each user was analyzed. Consequently, intra-individual variability and the reproducibility of repeated measurements could not be assessed. Fourthly, age, sex, and BMI are incorporated into the SW-PWV estimation algorithm. Therefore, the associations between SW-PWV and these variables are not independent of the measurement algorithm and should be interpreted primarily as descriptive characteristics of the algorithm-derived output rather than as evidence of independent biological effects on arterial stiffness. Validation against independently measured cfPWV is required to clarify these biological relationships. Fifthly, completion of the health questionnaire was voluntary, and the characteristics of questionnaire completers and non-completers could not be compared. Therefore, questionnaire response bias cannot be excluded, and the questionnaire subgroup may not be fully representative of the overall study population. Sixthly, sex, age, height, and weight were self-reported rather than objectively measured. Systematic under- or over-reporting of height and weight may therefore have introduced error into BMI estimation and affected the observed associations between BMI and SW-PWV. The diagnosis of CVDs was based on a questionnaire. Although the diseases were clearly defined, there is no guarantee that all the questionnaire results were entirely objective. The self-reported disease information was not verified. Therefore, recall bias and disease misclassification cannot be excluded. Some users may have forgotten previous diagnoses, misunderstood the questionnaire items, or been unaware of undiagnosed conditions. Underdiagnosis may have been particularly relevant among younger users or those with lower health awareness. These errors may have resulted in under- or overestimation of disease prevalence and may have attenuated or otherwise distorted the observed correlation coefficients between SW-PWV and cardiovascular conditions. Accordingly, these associations should be interpreted with caution. Seventhly, important potential confounders, including blood pressure, antihypertensive treatment, smoking, physical activity, and socioeconomic status, were not collected and could not be adjusted for. Therefore, the disease-related associations and RRs were unadjusted, and residual confounding cannot be excluded. Eighth, the proportion of stroke smartwatch wearers was very low, resulting in relatively limited data on stroke cases, which may influence the analysis results. Finally, because this was a cross-sectional study, causal relationships cannot be inferred, and the observed associations should not be interpreted as evidence of prospective risk prediction.

### 4.4 Future directions

Future studies should include repeated SW-PWV measurements from the same individuals to assess intra-individual variability and measurement reproducibility. Prospective longitudinal studies are also needed to determine whether SW-PWV is associated with subsequent cardiovascular outcomes. In addition, validation in more diverse and representative populations is warranted to improve the generalizability of the findings.

## 5 Conclusions

PWV measured by smartwatch was correlated with age, sex, and BMI, with varying correlation patterns across age groups. Individuals with hypertension, diabetes, dyslipidemia, or CVD exhibited elevated SW-PWV levels. In this cross-sectional study, an attempt to apply the cfPWV threshold 10 m/s to SW-PWV showed that SW-PWV ≥10 m/s was associated with a higher prevalence of CVD, suggesting that the cfPWV threshold 10 m/s recommended by the guidelines might serve as a preliminary or exploratory threshold reference for SW-PWV, pending direct validation against conventional cfPWV and confirmation in prospective longitudinal studies. Rather than replacing standard clinical assessment, SW-PWV could potentially be incorporated into existing care pathways as an initial community-based screening tool to identify individuals who may benefit from formal cardiovascular evaluation.

## Data Availability

The original contributions presented in the study are included in the article/Supplementary Material, further inquiries can be directed to the corresponding author/s.

## Supplementary material

Supplementary Figure S1 **Distribution of SW-PWV before and after individual-level outlier screening.** (**a**) Distribution of SW-PWV before outlier screening (n = 185,712). (**b**) Distribution of SW-PWV after outlier screening (n = 166,242). (**c**) Distribution of SW-PWV among the 19,470 users excluded according to the individual-level outlier criterion. The red dashed line indicates the mean, and the green dashed lines indicate the mean ± 3 standard deviations (SDs) of the distribution shown in each panel. SW-PWV, smartwatch-measured pulse wave velocity; SD, standard deviation.

Supplementary Figure S2 **Age–stratified ROC curves of SW–PWV for discriminating prevalent hypertension.** ROC curves are shown for participants aged (**a**) <30 years, (**b**) 30–39 years, (**c**) 40–49 years, (**d**) 50–59 years, (**e**) 60–69 years, and (**f**) ≥70 years. The red point indicates the optimal probability cutoff determined by the maximum Youden index. The dashed diagonal line represents discrimination no better than chance. AUC, area under the receiver operating characteristic curve; ROC, receiver operating characteristic; SW–PWV, smartwatch–measured pulse wave velocity.

Supplementary Figure S3 **ROC curve of SW-PWV for prevalent cardiovascular disease. (a)** ROC curve with the prespecified SW-PWV threshold of 10 m/s indicated (Sensitivity = 13.6%, Specificity = 96.7%). **(b)** ROC curve showing both the exploratory optimal threshold (8.53 m/s; Sensitivity = 56.5%, Specificity = 76.9%) and the prespecified 10 m/s threshold (Sensitivity = 13.6%, Specificity = 96.7%). Abbreviations: AUC, area under the curve; CVD, cardiovascular disease; ROC, receiver operating characteristic; SW-PWV, smartwatch-measured pulse wave velocity.
